# Neuroanatomical Correlates of Impulsive Choices and Risky Decision Making in Young Chronic Tobacco Smokers: A Voxel-Based Morphometry Study

**DOI:** 10.3389/fpsyt.2021.708925

**Published:** 2021-08-30

**Authors:** Aldo Alberto Conti, Alexander Mario Baldacchino

**Affiliations:** Division of Population and Behavioral Science, University of St. Andrews School of Medicine, St. Andrews, United Kingdom

**Keywords:** neuropsychology, nicotine, impulsivity, neuroimaging, tobacco, addictions, adolescents

## Abstract

**Introduction:** Impairments in the multifaceted neuropsychological construct of cognitive impulsivity are a main feature of chronic tobacco smokers. According to the literature, these cognitive impairments are relevant for the initiation and maintenance of the smoking behavior. However, the neuroanatomical correlates of cognitive impulsivity in chronic smokers remain under-investigated.

**Methods:** A sample of 28 chronic smokers (mean age = 28 years) not affected by polysubstance dependence and 24 matched non-smoker controls was recruited. Voxel Based Morphometry (VBM) was employed to assess Gray Matter (GM) volume differences between smokers and non-smokers. The relationships between GM volume and behavioral manifestations of impulsive choices (5 trial adjusting delay discounting task, ADT-5) and risky decision making (Cambridge Gambling Task, CGT) were also investigated.

**Results:** VBM results revealed GM volume reductions in cortical and striatal brain regions of chronic smokers compared to non-smokers. Additionally, smokers showed heightened impulsive choices (*p* < 0.01, Cohen's *f* = 0.50) and a riskier decision- making process (*p* < 0.01, Cohen's *f* = 0.40) compared to non-smokers. GM volume reductions in the left Anterior Cingulate Cortex (ACC) correlated with impaired impulsive and risky choices, while GM volume reductions in the left Ventrolateral Prefrontal Cortex (VLPFC) and Caudate correlated with heightened impulsive choices. Reduced GM volume in the left VLPFC correlated with younger age at smoking initiation (mean = 16 years).

**Conclusion:** Smokers displayed significant GM volume reductions and related cognitive impulsivity impairments compared to non-smoker individuals. Longitudinal studies would be required to assess whether these impairments underline neurocognitive endophenotypes or if they are a consequence of tobacco exposure on the adolescent brain.

## Introduction

Tobacco smoking is the leading cause of preventable death worldwide (1). According to the World Health Organization (WHO) (1), ~7 million people die each year because of direct tobacco smoking, while 1.2 million individuals die because of second-hand smoke. Despite recent trends showing a decrease in tobacco smoking in several developed countries, the percentage of tobacco smokers worldwide remains dramatically high (1). In fact, it is estimated that there are ~1.3 billion current tobacco smokers, 80% of these living in low and middle-income countries (1).

Notably, impairments in the neuropsychological domain of “cognitive impulsivity” constitute one of the main features of substance users (2–5), including chronic tobacco smokers (6–8). Particularly, impairments in cognitive impulsivity are considered relevant for the initiation and chronicity of substance use (5). Cognitive impulsivity is characterized by the cognitive subdomains of choice impulsivity (delay discounting) and risky decision making (2, 4). Choice impulsivity refers to the tendency to opt for immediate pleasures/rewards (e.g., drug of abuse) over long- term gains (e.g., long-term health) (4), while “risky decision making” refers to a type of decisional process occurring when a an individual engages in decisions despite the risk of suffering known adverse consequences. Both cognitive impaired processes are strongly predictive of treatment outcomes for substance use and dependence, including tobacco smoking cessation treatments (8, 9). In fact, tobacco smokers displaying heightened impulsive choices (high delay discounting rates) are likely to relapse and to jeopardize cessation attempts (10, 11).

Advancements in neuroimaging techniques and lesion studies have helped to determine the neurobiological correlates of the multifaceted nature of cognitive impulsivity in healthy individuals by associating functional and structural disruptions in (a) the Ventrolateral PreFrontal Cortex (VLPFC), Dorsolateral PreFrontal Cortex (DLPFC), and lateral OrbitoFrontal Cortex (lOFC) to heightened impulsive choices, and (b) deficits in the Anterior Cingulate Cortex (ACC), Ventromedial PreFrontal Cortex (VMPFC), and medial/rostral OrbitoFrontal Cortex (mOFC) to impaired risky decision making (12). Additionally, disruptions in the ventral and dorsal striatum (caudate and putamen) have been related to both impaired impulsive and risky choices (12). According to the competing neurobehavioral decision systems theory of addiction (13) (CNDS), an imbalance between the reward system (comprising the striatum, midbrain, amygdala, and insula) and the executive decision system (comprising medial and lateral prefrontal brain regions) may explain the cognitive impulsivity impairments commonly manifested by substance users. Specifically, a hyperactive impulsive system and a hypoactive executive decision system may cause a reward bias and a lack of self-control toward the substance of abuse.

Notably, studies conducted on polysubstance users who were tobacco smokers in addition to being dependent to other substances such as cocaine (14), alcohol (15), and opioids (16), all revealed an association between Gray Matter (GM) volume reductions in fronto- cortical and striatal brain regions and behavioral manifestations of cognitive impulsivity. Despite studies conducted in the last decade revealing GM volume reductions in fronto-cortical and striatal brain regions of chronic tobacco smokers not affected by polysubstance dependence (17–19), the relationship between these GM volume reductions and impairments in cognitive impulsivity in chronic smokers remains under-investigated and limited to self-reported measures of impulsivity (20). To our knowledge, only one study conducted by Durazzo et al. (21) investigated the relationship between GM thickness and behavioral manifestations of cognitive impulsivity through a decision making task (Iowa gambling task, IGT) in 41 chronic smokers with a mean age of 46 years (21). Results of this study revealed an association between poorer decision making and reduced cortical thickness in the ACC and VMPFC of chronic smokers. However, no association between structural brain deficits in frontal brain regions and impulsive choices (delay discounting) was investigated, nor between reduced cortical thickness and decision making under risk outside a learning context (as assessed by the Cambridge Gambling Task, CGT) (22).

Therefore, the aim of the current study was to test the following hypotheses:

1) Chronic tobacco smokers not affected by polysubstance dependence, neurodegenerative conditions and/or psychiatric illnesses display GM volume reductions compared to non-smoker controls in *a priori* brain regions of interest such as VLPFC, lOFC, DLPFC, VMPFC, mOFC, ACC, insula, dorsal (caudate and putamen) and ventral striatum (globus pallidus, thalamus) in contrast to non-smokers healthy controls. *A priori* regions of interest were determined based on previous meta-analyses investigating GM volume reductions in chronic smokers compared to non-smokers, and on previous studies/reviews investigating the neuroanatomical (GM) correlates of cognitive impulsivity in substance users (studies are listed in Supplementary Tables 1a, 1b).
2) Chronic tobacco smokers display impairments in impulsive choices and risky decision-making compared to non-smokers as assessed by computerized measures of cognitive impulsivity such as the five- trial Adjusting Delay Discounting Task (ADT-5) (23) and the Cambridge Gambling Task (CGT) (22).
3) GM volume reductions in *a priori* regions of interest are correlated to heightened impulsive choices and impaired risky decision making in chronic smokers.

## Materials and Methods

### Recruitment

Community based chronic tobacco smokers and non-smokers were recruited through a convenient-sampling approach across the South Eastern regions of Scotland between October 2019 and March 2020. Different methods of recruitment were employed, these included: Internet advertisements posted on Gumtree and Craiglist websites, flyers distributed at local businesses (e.g., supermarkets, leisure centers), advertisements published on “The Courier” regional newspaper, and word of mouth. All participants provided written informed consent prior to the beginning of the study. They were rewarded a total of £100 for their full participation. The recruitment flow chart is depicted in Supplementary Figure 1.

Enrolled participants needed to attend two experimental sessions. These sessions were conducted on separate days within the same week (no more than 3 days apart): The first experimental session involved screening procedures and computerized measures of cognitive impulsivity (impulsive choices and risky decision making). This experimental session was conducted at the University of St Andrews School of Medicine in St Andrews. The second session involved a Magnetic Resonance Imaging (MRI) procedure at the Clinical Research Center (CRC), Ninewells Hospital, Dundee.

### Inclusion and Exclusion Criteria

Participants were screened for eligibility (see Supplementary Table 2) through objective and subjective measurements. Specifically, an exhaled Carbon Monoxide (CO) test was utilized to measure CO levels in participants' breath, while a saliva drug testing kit was employed to determine presence (or not) for cotinine. The presence of cannabis, cocaine, morphine, methadone, amphetamine, methamphetamine, and benzodiazepines was assessed through a urine drug screening test.

Chronic tobacco smokers who reported a recreational use of cannabis (no more than twice per week) mixed with tobacco in the same “spliff” or “joint,” and did not present symptoms of acute intoxication (e.g., conjunctival injection, slurred speech, agitation) at the screening session, were not excluded from the study. In fact, the effect of recreational cannabis use on cortical brain structures is limited (24), whereas the effect of cannabis use on subcortical structures may be better explained by concomitant tobacco smoking (25). Meta-analytic findings also revealed a small effect of cannabis use on cognitive impulsivity with an effect size of 0.30 (26). The Mini International Neuropsychiatric Instrument (MINI) version 7.0.2 (27) was utilized to exclude the presence of DSM V psychiatric disorders (Axis I) for participants. A screening interview was utilized to exclude the presence of chronic conditions (e.g., HIV, diabetes), pregnancy, neurological disorders, and severe head injuries. Participants' patterns of tobacco use, smoking variables (pack years, cigarettes smoked per day, age at regular smoking onset), and weekly use of cannabis and alcohol were assessed through a paper and pencil questionnaire, while severity of nicotine dependence was assessed by using the Fagerström Test for Nicotine Dependence (FTND) (28). Participants' pre- morbid IQ was estimated through the Barona equations (29).

### Instruments

#### Cognitive Measures

During this experimental session participants needed to complete two computerized measures of cognitive impulsivity: The 5 trial Adjusting Delay Discounting Task (ADT-5) (23) and the Cambridge Gambling Task (CGT) (22).

The ADT-5 measures choice impulsivity. Participants were presented with choices between two fixed hypothetical monetary rewards (£5 available immediately and £10 available at some time in the future) over five trials. The delay period was adjusted after each trial based on participants' previous choice. Outcome measures for this task consisted in Effective Delay 50% (ED50) values. ED50 represents “the delay that is effective in discounting the subjective value of the delayed reinforcer by 50%” 30. It consists in the inverse of the discounting rate k (ED50 = 1/k) (30).

The Cambridge Gambling Task (CGT) is a computerized measure of risky decision making outside a learning context (22) that is part of the Cambridge Neuropsychological Test Automated Battery (https://www.cambridgecognition.com/cantab/cognitive-tests/executive-function/cambridge-gambling-task-cgt/). During this task, a yellow token was hidden either in red or blue boxes over five stages. Participants needed to guess the location of the token by selecting a proportion of points to bet on their decision. Each participant started with 100 points. Participants lost or gained points depending on their choices. Specifically, participants gained points for each correct guess and lost points for each incorrect guess. The amount of points gained or lost consisted in the amount of points betted by the participants. Outcome domain measures for this task consisted in the multiple facets of the risky decision-making process commonly manifested by substance users (6, 16, 31). Specifically, outcome measures consisted in (a) the average number of points betted after choosing the most likely outcome (risk taking score); (b) the overall proportion of points betted during the task (overall proportion bet score) [both (a) and (b) outcome measures reflect a propensity toward risk]; (c) the proportion of all trials where the subject chose the majority box color, which reflects the rationality of the participants' decision making process (quality of decision making score); and (d) the ability to modify choices in light of information about the probability of different outcomes (risk adjustment score) (6, 16, 31).

#### Neuroimaging

Structural T1 weighted images were acquired through a Siemens 3T Prisma-FIT scanner (Siemens Healthineers, Erlangen, Germany). Specifically, an MP-RAGE (magnetization-prepared rapid acquisition gradient echo) sequence was utilized to acquire images with a voxel size 0.8 × 0.8 × 1.0 mm^3^ with whole brain coverage, repetition time (TR) = 1.9 s, echo time (TE) = 2.64 ms. Flip angle = 9°, FOV = 200 mm, matrix = 256 × 256, 176 slices, slice thickness 1 mm. Scans were reported by a consultant radiologist to rule out the presence of incidental findings.

### Statistical Analysis

Data obtained from computerized measures of cognitive impulsivity (ADT-5, CGT) were analyzed through analyses of covariance (ANCOVAs) to test the null hypothesis of no differences between chronic tobacco smokers and non-smokers in relation to impulsive choices and risky decision-making outcome measures while controlling for relevant sociodemographic variables such as sex, age, premorbid IQ, educational level, social deprivation (SIMD), and other substances used (cannabis, alcohol). To be analyzed through ANCOVAs, data needed to meet the assumptions of normality, homoscedasticity, homogeneity of variances, and of homogeneity of regression slopes. Data failing assumption of normality and/or homogeneity of variances were Log10 transformed. The non-parametric Kruskall-Wallis H test was employed if data still violated assumptions of normality and/or of homogeneity of variances after transformation. Bonferroni-corrected pairwise comparisons with a significance level set at *p* < 0.05 were utilized to control for Type-1 error. SPSS v. 26 (SPSS Inc., USA) was utilized for this part of the analysis. Effect sizes (Choen's *f*) were computed through the software G^*^Power.

Neuroimaging data were analyzed by applying a whole-brain voxel-based morphometry (VBM) technique through SPM12 (https://www.fil.ion.ucl.ac.uk/spm/). T1 weighted images were segmented into gray and white matter probability maps, these were subsequently normalized to the Montreal Neurological Institute (MNI) template. Modulation was performed with Jacobian determinants to preserve the total amount of GM and WM in each probability map. Following segmentation and spatial normalization, images were smoothed with an 8 mm Gaussian kernel (32). Total intracranial volume was estimated by the Computational Anatomy Toolbox (http://www.neuro.uni-jena.de/cat/).

A two sample *T*-tests was utilized to assess for GM volume differences between chronic tobacco smokers and non-smokers across the whole brain, including *a priori* regions of interest (Supplementary Tables 1a, 1b). Whole-brain voxel-wise linear regression models were also computed to investigate the relationship between GM volume reductions and cognitive impulsivity outcome measures (ADT-5, CGT) in chronic tobacco smokers. Proof of concept analyses were conducted by computing whole-brain voxel-wise linear regression models investigating the relationship between GM volume reductions and tobacco exposure variables (cigarettes smoked per day, pack years, FTND, age at regular smoking onset).

Brain regions (including *a priori* regions of interest) were identified by converting MNI coordinates into Talairach coordinates (https://bioimagesuiteweb.github.io/webapp/mni2tal.html), and by inserting them into the Talairach Daemon Atlas (http://www.talairach.org/daemon.html) (16). A cluster forming significance threshold of *p* < 0.05 with a minimum of 100 contiguous voxels (k) per cluster was employed for two sample *T*-tests and for voxel-wise regression models testing the associations between GM volume and cognitive impulsivity measures. This threshold was obtained by applying Monte Carlo simulations (16, 33). For proof of concept analyses a more stringent threshold of *p* < 0.01 with a minimum of 100 contiguous voxels per cluster was employed. Covariates of no interest consisted in total intracranial volume (TIV), age, and biological sex for two sample *T*-tests and linear regression models. Results of whole-brain voxel-wise analyses are only reported in the text for *a priori* regions of interest.

## Results

### Sociodemographic and Smoking Characteristics

Chronic tobacco smokers and non-smokers were well-matched for age, Scottish index of multiple deprivation (SIMD) scores, and units of alcohol consumed per day at the time of the first experimental session as assessed by independent samples *t*-tests (*p* > 0.05). Chi-square test results did also reveal the absence of a statistically significant association between sex and smoking status, ${\text{χ}}_{\left(1\right)}^{2}$ = 0.55, *p* = 0.46. However, chronic tobacco smokers displayed lower pre- morbid IQ and a lower educational level compared to non-smokers (*p* < 0.05). Sociodemographic and tobacco smoking characteristics of participants are displayed in Table 1. At the time of the second experimental session (MRI), chronic smoker participants were still matched for sex, age, SIMD, and units of alcohol consumed per day to non-smoker controls (Table 1). No significant changes in smoking characteristics were detected between the two sessions (paired samples *t*-test *p* > 0.05).

**Table 1:** Sociodemographic characteristics, smoking characteristics, and cognitive impulsivity scores of study participants.

	**Session 1**			**Session 2**		
	**Chronic tobacco smokers**	**Non-smokers**	**Sig^**1**^**	**Chronic tobacco smokers**	**Non-smokers**	**Sig^**1**^**
**Sociodemographic characteristics**						
*n*	28	24		23	19	
Age in yrs (SD)	28.1 (8.3)	28.5 (9.5)	*p* = 0.96	28.5 (8.3)	29.7 (9.8)	*p* = 0.66
Sex (%)	64.3% Males 35.7% Females	54.2% Males 45.8% Females	*p* = 0.45	60.9% Males 39.1% Females	57.9% Males 42.1% Females	*p* = 0.54
Level of education (SD)	3.5 (0.7)	4.2 (0.6)	NS > CS = *p* < 0.01	3.4 (0.8)	4.2 (0.7)	NS > CS = *p* < 0.01
Pre-morbid IQ (SD)	102.8 (3.6)	107.5 (4.1)	NS > CS = *p* < 0.001	102.4 (3.7)	107.9 (4.3)	NS > CS = *p* < 0.001
SIMD (SD)	2.0 (1.5)	2.6 (1.4)	*p* = 0.12	1.7 (1.3)	2.4 (1.5)	*p* = 0.13
**Tobacco smoking characteristics**						
Cigarettes smoked per day	15.0 (4.5)	N/A	N/A	15.5 (4.2)	N/A	N/A
FTND	5.0 (1.5)	N/A	N/A	5.1 (1.5)	N/A	N/A
Pack years	10.4 (8.1)	N/A	N/A	11.2 (8.3)	N/A	N/A
Age at regular smoking onset in yrs*	16.1 (3.4)	N/A	N/A	16.0 (3.7)	N/A	N/A
CO level	21.8 (1.2)	1.2 (0.5)	CS > NS = *p* < 0.001	N/A	N/A	N/A
**Other substances used**						
Units of alcohol consumed x day (SD)	0.6 (1.0)	0.2 (0.5)	*p* = 0.16	0.5 (0.8)	0.2 (0.3)	*p* = 0.11
*n* cannabis smokers	5	N/A	N/A	4	N/A	N/A
**Cognitive impulsivity scores**						
ADT-5**ED50	29.50 (39.22)	434.45 (922.79)	NS > CS *p* = < 0.01	29.58 (38.16)	517.78 (102.69)	NS > CS = *p* < 0.01
CGT risk taking	0.64 (0.17)	0.47(0.18)	CS > NS = *p* < 0.01	0.64 (0.15)	0.47 (0.19)	CS > NS = *p* < 0.01
CGT overall proportion bet	0.60 (0.17)	0.43 (0.17)	CS > NS = *p* < 0.01	0.60 (0.15)	0.42 (0.18)	CS > NS = *p* < 0.01
CGT risk adjustment	1.30 (1.11)	1.79 (1.30)	*p* = 0.63	1.46 (1.19)	1.70 (1.37)	*p* = 0.72
CGT quality of decision making	0.94 (0.85)	0.93 (0.10)	*p* = 0.20	0.90 (0.13)	0.92 (0.11)	*p* = 0.23

*A mean of 2.8 days separated session 1 from session 2. Data are presented in Means and Standard Deviations (SD) or in percentages (%). Sig^1^ = significance at p < 0.05 two tailed. Education level scores (1 = Left formal education before age 16, 2 = Left formal education at age 16, 3 = Left formal education at age 18, 4 = Undergraduate degree, 5 = Master's degree/Post-Graduate Diploma, 6 = PhD). %, percentage; SIMD, Scottish index of Multiple Deprivation (1 = most deprived area to 5 = least deprived area); n, number of participants; NS, non-smokers; CS, chronic tobacco smokers; CO, Carbon Monoxide. Yrs, years; SD, Standard Deviation; FTND, Fagerström Test for Nicotine Dependence (0–2 = very low dependence, 2–4 = low dependence, 5 = medium dependence, 6 or more = high dependence). ADT-5, 5 trial adjusting delay discounting task; ED50, Effective delay 50; CGT, Cambridge Gambling task*.

* *Age at regular smoking onset was defined as the age at which individuals started smoking 5 or more cigarettes daily*.

** *ADT-5 scores (ED50 values) were log10 transformed for ANCOVA analyses*.

### Cognitive Tests

#### 5-Trial Adjusting Delay Discounting Task

A statistically significant difference was detected between chronic tobacco smokers and non-smokers in relation to ED50 values [*F*_(1,44)_ = 11.39, *p* < 0.01, partial η^2^ = 0.200,Cohen's *f* = 0.50] after adjusting for age, premorbid IQ, level of education, sex, units of alcohol consumed per day, occasional cannabis use, and SIMD covariates. Pairwise comparisons with Bonferroni adjustment revealed that non-smokers had significantly higher ED50 values (*M* = 1.89, SE = 0.21) compared to chronic smokers (*M* = 0.82, SE = 0.19) with a mean difference of 1.07 (95% CI, 0.43–1.71) *p* < 0.01. These results indicated that the delay at which £10 had lost 50% of its value (ED50) was longer for non-smokers compared to chronic smokers.

#### Cambridge Gambling Task

A statistically significant difference was also detected between chronic smokers and non-smokers in relation to “risk taking” scores [*F*_(1,44)_ = 6.61, *p* < 0.01, partial η^2^ = 0.133, Cohen's *f* = 0.39] and “overall proportion bet” scores [*F*_(1,44)_ = 7.28, *p* < 0.01, partial η^2^ = 0.153, Cohen's *f* = 0.42] after adjusting for relevant covariates. Pairwise comparisons with Bonferroni adjustment revealed that chronic smokers had significantly higher “risk taking” scores (*M* = 0.64, SE = 0.04) compared to non-smokers (*M* = 0.47, SE = 0.04) with a mean difference of 0.172 (95% CI, 0.03–0.30) *p* < 0.01. Similarly, chronic smokers had significantly higher “overall proportion bet” scores (*M* = 0.59, SE = 0.03) compared to non-smokers (*M* = 0.42, SE = 0.04) with a mean difference of 0.173 (95% CI, 0.04–0.30) *p* < 0.01. No statistically significant difference was found between chronic smokers and non-smokers in relation to “risk adjustment” scores [*F*_(1,44)_ = 0.22, *p* = 0.63, partial η^2^ = 0.00]. Finally, no statistically significant difference was identified between chronic smokers and non-smokers in relation to “quality of decision making” scores as assessed by a Kruskall Wallis H test [*H*_(1)_ = 1.63, *p* = 0.20].

*post-hoc* power calculations with a sample size of 52 participants and α = 0.05 revealed a power (1-β probability) of 0.94 for choice impulsivity (ADT-5) scores, a power of 0.84 for “overall proportion bet” scores (CGT), and a power of 0.78 for “risk taking” scores (CGT).

Considering the dropout of 10 participants (5 smokers and 5 non-smokers) prior to the MRI session, sensitivity analyses (ANCOVAs) were conducted to ascertain choice impulsivity (ADT-5) and risky decision making (CGT) differences between the remaining smokers (*n* = 23) and non-smokers (*n* = 19). Results still revealed a significant difference (*p* < 0.01) between smokers and non-smokers in relation to ED50 values, “risk taking,” and “overall proportion bet” scores with effect sizes (Cohen's *f*) of 0.57, 0.40, and 0.42 respectively.

### Neuroimaging

Voxel based morphometry (VBM) results revealed GM volume reductions in chronic tobacco smokers compared to non-smokers in several cortical and striatal brain regions across the whole brain, predominantly in the left hemisphere. Structures presenting GM volume reductions, cluster sizes, Brodmann's areas (BA), and corresponding Montreal Neurological Institute (MNI) coordinates are reported in Supplementary Table 3. Regions of Interest displayed GM volume reductions in frontal cortices such as the bilateral ACC (14, 21, 29;−11, 26, 18; BA32), left DLPFC (−21, 41, 23; BA9), left VLPFC (−26, 35,−12; BA47), and left OFC (−5, 44, −27; BA11). Striatal gray matter reductions in chronic smokers compared to non-smokers were present in the left caudate (−11, 2, 12), and in the left putamen (−21, −9, 6).

### Relationship Between Neuroimaging and Non-neuroimaging Measures

**Figure 1:**
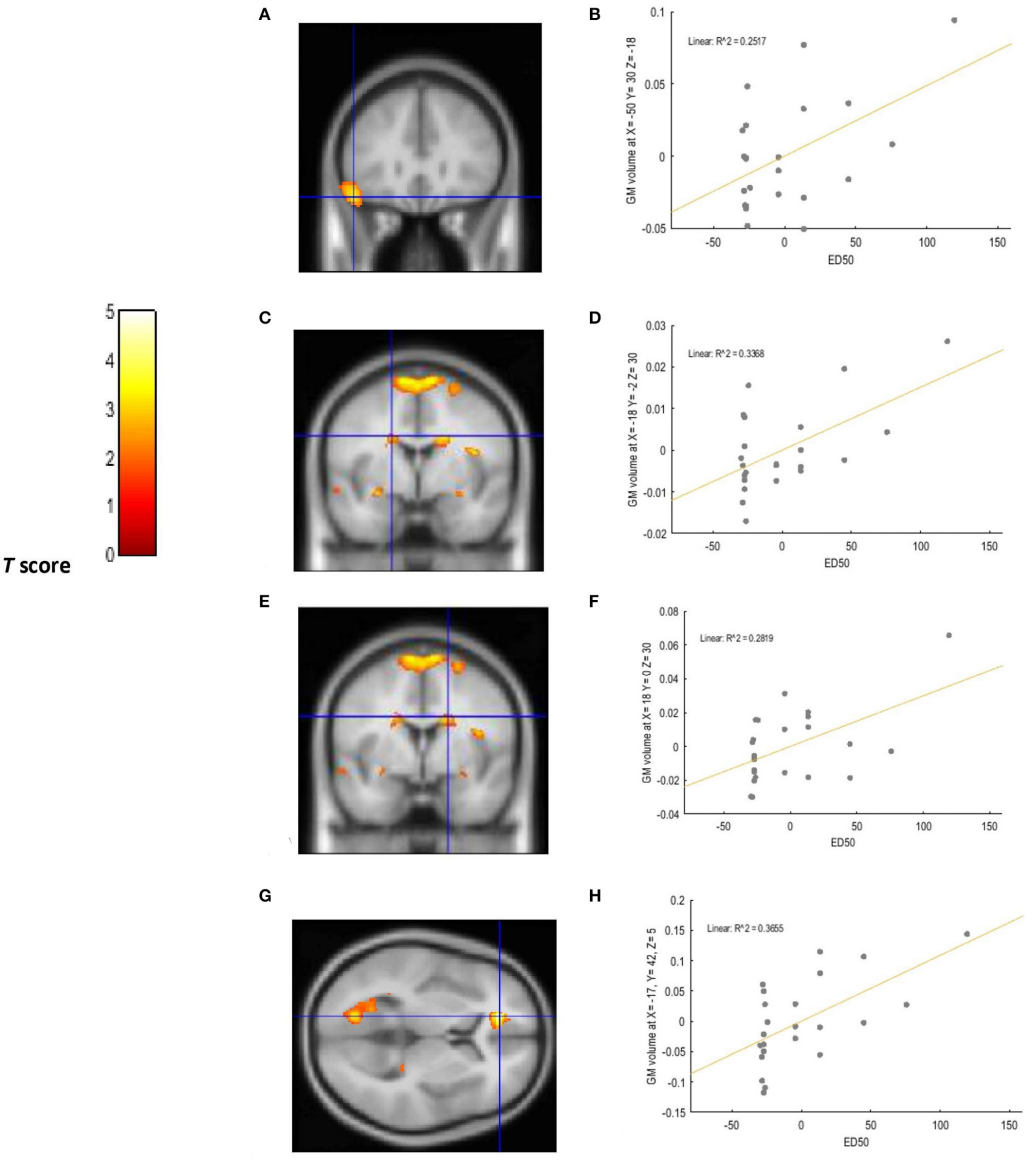
Voxel-wise regression results depicting positive relationships between ED50 values and GM volume in the left VLPFC, left ACC, and bilateral caudate while controlling for total intracranial volume (TIV), age, and biological sex. The cluster-forming threshold consisted in *p* < 0.05 with a minimum of 100 contiguous voxels per cluster. The figure shows the location of the left VLPFC **(A)**, left caudate **(C)**, and right caudate **(E)** voxel clusters in coronal slices. The location of the left ACC voxel cluster **(G)** is depicted in an axial slice. For visualization purposes, the scatterplot of adjusted response data **(B)** shows the significant (*p* < 0.005) reduction in GM volume as a function of ED50 values in the left VLPFC (cluster size: 1624 voxels). The scatterplot of adjusted response data **(D)** shows the significant (*p* < 0.005) reduction in GM volume as a function of ED50 values in the left caudate (cluster size: 469 voxels). The scatterplot of adjusted response data **(F)** shows the significant (*p* < 0.005) reduction in GM volume as a function of ED50 values in the right caudate (cluster size: 728 voxels). The scatterplot of adjusted response data **(H)** shows the significant (*p* < 0.005) reduction in GM volume as a function of ED50 values in the left ACC (cluster size: 732 voxels). Plotted values consist in residuals of mean GM volume (Y axis) and of mean ED50 values (X axis). *R*^2^: Coefficient of determination.

**Figure 2:**
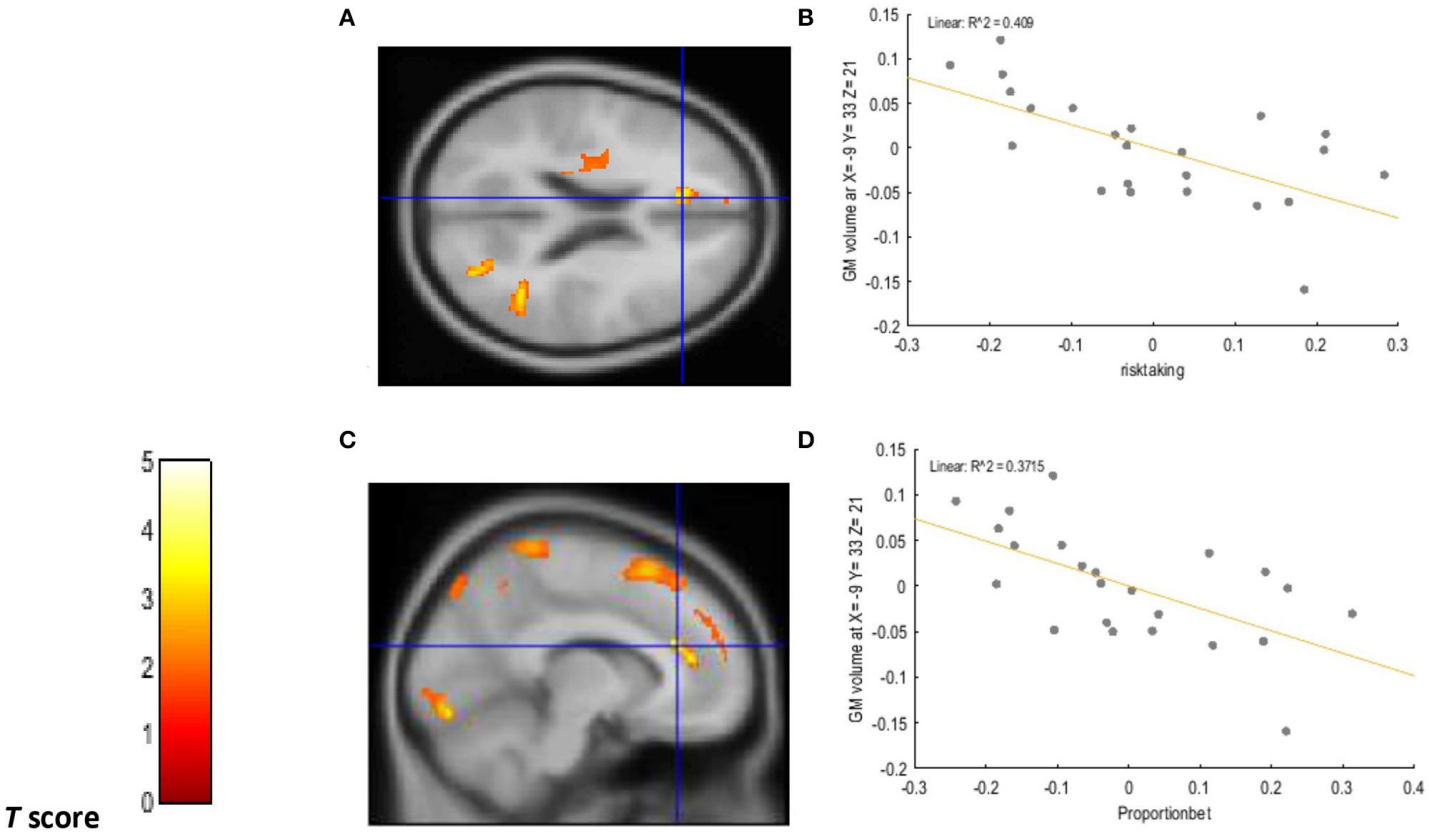
Voxel-wise regression results depicting negative relationships between risk taking scores, overall proportion bet scores, and GM volume in the left ACC while controlling for total intracranial volume (TIV), age, and biological sex. The cluster-forming threshold consisted in *p* < 0.05 with a minimum of 100 contiguous voxels per cluster. The figure shows the location of the left ACC voxel clusters in axial **(A)** and coronal **(C)** slices. For visualization purposes, the scatterplot of adjusted response data **(B)** shows the significant (*p* < 0.005) reduction in GM volume as a function of risk-taking scores in the left ACC (cluster size: 256 voxels). The scatterplot of adjusted response data **(D)** shows the significant (*p* < 0.005) reduction in GM volume as a function of overall proportion bet scores in the left ACC (cluster size: 227 voxels). Plotted values consist in residuals of mean GM volume (Y axis) and of mean risk taking and overall proportion bet scores (X axis). *R*^2^: Coefficient of determination.

A positive relationship was detected between choice impulsivity scores (ED50 values) and GM volume in regions of interest. These included the left VLPFC (−50, 30, −18; BA47) (*p* = 0.002; *T* = 3.20; *R*^2^ = 0.251), left caudate (−18, −2, 30) (*p* = 0.002; *T* = 3.21; *R*^2^ = 0.336), right caudate (18, 0, 30) (*p* = 0.002; *T* = 3.37, *R*^2^ = 0.281), and left ACC (−17, 42, 5, BA32) (*p* < 0.0001; *T* = 4.57, *R*^2^ = 0.365) of chronic tobacco smokers. These associations are depicted in Figure 1. Furthermore, a negative relationship was detected between “risk taking” (*p* =0.001; *T* = 3.62; *R*^2^ = 0.409) and “overall proportion bet” (*p* = 0.002; *T* = 3.34; *R*^2^ = 0.371) scores of the CGT and GM volume in the left ACC (−9, 33, 21; BA 32) of chronic tobacco smokers (see Figure 2). Supplementary Tables 4, 5 depict the associations between risky decision making, impulsive choices and GM volume in brain regions of no interest (not related to the *a priori* hypotheses listed in the section Introduction).

Negative and positive relationships were also detected between GM volume in regions of interest and smoking variables. These are depicted in Table 2. Regression plots showing the directions of these associations are illustrated in Supplementary Figure 2. Supplementary Table 6 illustrates the associations between tobacco smoking variables and GM volume in brain regions of no interest.

**Table 2:** Voxel-wise regression results depicting significant associations between GM volume in regions of interest and smoking variables while controlling for TIV, age, and biological sex.

**Covariate of interest**	**Hemisphere**	**BA**	**MNI coordinates (x, y, z)**	**Peak *T-*values**	***P-*values**	**Cluster size (*k*)**	***R*^**2**^**	**Region of interest**
Age at regular smoking onset	L	9	−29, 32, 32	3.36	*p* < 0.005	125	0.341	DLPFC
	L	47	−39, 30, −2	2.86	*p* < 0.005	116	0.274	VLPFC
Pack years	L	8	−14, 33, 39	4.81	*p* < 0.0001	474	0.211	DLPFC
	L	N/A	−24, 3, 5	4.10	*p* < 0.0001	174	0.183	Putamen
	L	10	−17, 54, 0	3.59	*p* < 0.005	105	0.154	ACC
	L	N/A	−9, 11, 6	3.56	*p* < 0.005	132	0.154	Caudate
	L	46	−41,21, 23	3.40	*p* < 0.005	335	0.146	DLPFC
Cigarettes smoked per day	L	N/A	−26, −2, 5	3.71	*p* < 0.005	195	0.284	Putamen
FTND	L	N/A	−15, −23, 5	3.25	*p* < 0.005	197	0.251	Thalamus
	L	47	−50, 42, −2	3.04	*p* < 0.005	125	0.226	VLPFC

*The Cluster forming threshold consisted in p < 0.01 (uncorrected) with a minimum of 100 contiguous voxels (k) per cluster. MNI, Montreal Neurological Institute; VLPFC, VentroLateral PreFrontal Cortex; DLPFC, Dorsolateral PreFrontal cortex; ACC, anterior cingulate cortex; T, t-statistics; BA, Brodmann's area*.

## Discussion

### Summary of Findings

a) Chronic tobacco smokers displayed GM volume reductions in various brain areas (predominantly in the left hemisphere of the brain), including striatal and cortical a priori regions of interest located in the prefrontal cortex, supporting hypothesis 1.
b) Chronic tobacco smokers displayed heightened impulsive choices and a riskier decision-making process in comparison to non-smoker controls as assessed by the ADT-5 and CGT tasks, supporting hypothesis 2.
c) GM volume reductions in cortical and striatal *a priori* regions of interest were correlated to impulsive choices (VLPFC, caudate, ACC) and risky decision making (ACC) in chronic tobacco smokers, supporting hypothesis 3.

### Interpretation

These results are in line with recent findings demonstrating a strong association between chronic tobacco smoking, impulsive choices, and risky decision making (6, 7). That is, chronic tobacco smokers have a greater difficulty in delaying immediate gratification and display a riskier decision-making process in comparison to non- smokers. The current study adds up to the body of knowledge by revealing a cross- sectional relationship between impulsive choices, risky decision making, and GM volume reductions in chronic tobacco smokers not affected by psychiatric comorbidity, poly substance dependence (e.g., alcoholism), and/or chronic medical conditions (e.g., HIV). These deficits were detected in a relatively young sample of cigarettes smokers with a mean age of 28 years old. Thus, it is unlikely that participants were affected by neurocognitive impairments that are a consequence of the aging process (34).

Under a neuroanatomical point of view, the above findings are in line with previous studies revealing GM volume reductions in striatal and cortical structures located in the prefrontal cortex of chronic tobacco smokers (17–19). In accordance with these studies, the current research revealed a negative relationship between tobacco exposure variables and reduced GM volume in prefrontal and striatal brain regions. Specifically, longer pack years were associated to reduced GM volume in the left DLPFC, left ACC, left caudate, and left putamen. Severity of nicotine dependence (FTND score) was also associated with GM volume reductions in the left thalamus, an area strictly related to the development and maintenance of substance dependence for its dopaminergic projections (35). The current study provides support to the findings of Durazzo et al. (21) by revealing a correlation between GM volume reductions in the left ACC and risky decision making in chronic smokers. Furthermore, the current study revealed a positive association between GM volume reductions in the left ACC and the inability to delay gratification displayed by chronic smokers (ED50 values). Indeed, the ACC has been proposed to play a crucial role in the maintenance of addictive behaviors due to its connections with the limbic system and prefrontal cortex, therefore mediating maladaptive emotional and reward-based decisions (36). The association between GM volume reductions in the VLPFC and heightened impulsive choices displayed by chronic smokers extends previous work by endorsing disruptions in brain's executive system as a core feature of nicotine dependent individuals (36).

Notably, impairments in cognitive impulsivity for chronic smokers enrolled in the current study correlated to GM volume reductions localized predominantly in the left hemisphere of the brain. Despite GM deficits being reported frequently in left fronto-cortical and striatal brain regions in chronic tobacco smokers (19, 37, 38), no association between cognitive impairments and lateralization of structural brain deficits in chronic smokers was ever investigated. However, an fMRI study conducted by Clewett et al. (39) revealed greater functional coupling between the left fronto- parietal network and the left insular cortex in chronic tobacco smokers compared to non-smokers while performing a computerized Delay Discounting task (with hypothetical monetary rewards) (39). Greater functional connectivity between these areas was also associated to steeper discounting rates (39). Furthermore, a systematic review conducted by Gordon (40) showed greater peak activation in fronto-cortical brain areas located in the left hemisphere of tobacco deprived smokers while responding to cue-reactivity (craving) stimuli during fMRI investigations (40). Therefore, consistently with the CNDS model, the simultaneous hyperactivation of the left reward system to cigarettes cues, and the inability to delay gratification caused by GM volume reductions in the left VLPFC and left ACC, may induce cigarettes smokers to crave for, and to want immediately the drug of abuse (i.e. cigarettes) despite the known health risks associated to its usage. Indeed, impulsivity has been linked to tobacco craving and smoking relapses by the literature (41).

Even though the cross-sectional nature of the current research does not allow to directly imply causation, the above findings may be explained by neuroscience- based paradigms. According to the “tobacco Induced neurotoxicity theory of adolescent cognitive development” (TINACD) (42), exposure to tobacco during adolescence (a developmental period characterized by intense neurostructural and neurochemical maturation) may cause long-lasting deficits in frontal brain areas modulating cognitive functions mostly related to top- down inhibitory control and decision making (42). For this reason, adult smokers who initiated tobacco use during adolescence may have more difficulties quitting smoking, and relapse more frequently, in comparison to individuals who started smoking at a later developmental stage (42). This theory has been availed by animal models revealing unique, and long- lasting, cellular alterations and structural changes in fronto-cortical and striatal brain regions of rats exposed to nicotine during adolescence (43, 44). In support to the TINACD, the current study revealed a positive correlation between younger age at regular smoking onset (mean = 16 years) and GM volume reductions in the left VLPFC (BA 47), (Table 2). As described previously, GM volume reductions in the left VLPFC (BA 47) were also associated to heightened impulsive choices in the current sample (Figure 1).

An alternative interpretation to the above findings consists in the presence neurocognitive endophenotypes in substance dependent populations (45). In fact, studies have shown abnormal brain structures in fronto-cortical and striatal brain regions, and related cognitive impulsivity impairments, in substance dependent individuals and their drug- naïve biological siblings (46, 47). However, because of the lack of longitudinal studies, the causal relationship between chronic tobacco smoking and neurocognitive impairments remains unclear (48).

### Clinical Relevance

Considering that impaired cognitive impulsivity may be determinant in fostering tobacco smoking during different stages of the drug addiction cycle (initiation, maintenance, relapse) (8, 11), results from this study may inform smoking cessation treatments, and neuroscience-based interventions for tobacco use and dependence. Specifically, the identification of GM volume reductions in brain areas related to different aspects of cognitive impulsivity (ACC to risky decision making, VLPFC, ACC, and caudate to impulsive choices), and the proposed left lateralization of these neurocognitive impairments, may aid the improvement of technology-based neuromodulation interventions such as transcranial direct current stimulation (tDCS) and transcranial magnetic stimulation (rTMS). In fact, such interventions have provided limited and contrasting results for the treatment of nicotine dependence (49).

Moreover, the inability to delay gratification, commonly featured by chronic smokers (7), should be considered a primary target for cognitive rehabilitation therapies (CRTs) (50). Studies have also proposed Episodic Future Thinking (ETF) interventions as effective means to decrease delay discounting rates, cigarette self- administration, and cigarette demand (51).

### Strengths and Limitations

The current study should be considered in light of several limitations. First, the size of the sample enrolled in the current study was relatively small, therefore limiting the power of risky decision making (CGT) results. However, *post-hoc* power calculations for choice impulsivity (ADT-5) results revealed an achieved power of 0.94 with a very large effect size of 0.5. Additionally, participants were all recruited from the same deprived metropolitan area, thus generalizability may be limited to Scottish chronic smokers from a low socio-economic background (SES). Studies have proposed a relationship between low SES, brain development deficits, and cognitive impairments (52). Although, chronic tobacco smoking has been recently found to mediate these associations (53). Another limitation consists in the utilization of self- reported questionnaires to assess alcohol consumption patterns and smoking characteristics of participants (pack years, cigarettes smoked per day, age at regular smoking onset) as participants may have provided inaccurate information. Nonetheless, a rigorous screening procedure involving different objective measurements to assess smoking status (exhaled CO, salivary cotinine) and to exclude the presence of other substances in participants' system (urine analysis) was employed. The recruitment of a younger chronic smoker sample not affected by polysubstance dependence and characterized by minimal alcohol and cannabis use, with the utilization of stringent exclusion and inclusion criteria, may be considered strengths of the current study.

### Conclusion

The current study revealed an association between left fronto-cortical and striatal GM volume reductions and impaired cognitive impulsivity in chronic tobacco smokers. GM volume reductions in the left VLPFC correlated to heightened impulsive choices and to younger age at regular smoking onset. Considering that the cross-sectional nature of the current research limits inferences of causal effects, longitudinal studies would be required to elucidate if these neurocognitive impairments are representations of pre-morbid endophenotypes or are caused by tobacco exposure on the developing adolescent brain.

## Data Availability Statement

The raw data supporting the conclusions of this article will be made available by the authors, without undue reservation.

## Ethics Statement

The studies involving human participants were reviewed and approved by National Health Service (NHS) London Bromley Research Ethics Committee (REC) (REC Reference Number: 19/LO/1176), and by the University of St. Andrews Teaching and Research Ethics Committee (UTREC) (UTREC Approval Code: MD14516). The patients/participants provided their written informed consent to participate in this study.

## Author Contributions

AC and AB were responsible for the study concept and design. AC contributed to the recruitment of participants, acquisition data, performed the analysis of data, and drafted the manuscript. AB supervised recruitment, screening procedures, assisted with data analysis, interpretation of findings, and provided critical revision of the manuscript for important intellectual content. All authors critically reviewed content and approved final version for publication.

## Conflict of Interest

The authors declare that the research was conducted in the absence of any commercial or financial relationships that could be construed as a potential conflict of interest.

## Publisher's Note

All claims expressed in this article are solely those of the authors and do not necessarily represent those of their affiliated organizations, or those of the publisher, the editors and the reviewers. Any product that may be evaluated in this article, or claim that may be made by its manufacturer, is not guaranteed or endorsed by the publisher.
