# Supplementary material for: Neuroanatomical Correlates of Impulsive Choices and Risky Decision Making in Young Chronic Tobacco Smokers: A Voxel-Based Morphometry Study
Source: Front Psychiatry. 2021 Aug 30;12:708925. doi: 10.3389/fpsyt.2021.708925 (PMC8435625; doi:10.3389/fpsyt.2021.708925)
Supplement: Supplementary file 1 [file Table_1.docx]

**Supplementary Table S1a.** Studies investigating GM volume differences between smokers and nonsmokers

| **Study** | **Study type** | **Objective** | **ROIs** |
| --- | --- | --- | --- |
| Yang et al (2020) | Meta-analysis of VBM studies | To assess GM volume differences between smokers and nonsmokers | OFC, ACC, DLPFC, INSULA, lingual  gyrus, occipital gyrus |
| Sutherland et al (2016) | Meta-analysis of VBM studies | To assess GM volume differences between smokers and nonsmokers | Thalamus, DMPFC, VMPFC, VLPFC, DLPFC, Para-hippocampal gyrus, OFC, Insula, Cerebellum |
| Zhong et al (2016) | Meta-analysis of VBM studies | To assess GM volume differences between chronic smokers and nonsmokers | ACC, DLPFC, OFC |
| Pan et al (2012) | Meta-analysis of VBM studies | To assess GM volume differences  between chronic smokers and nonsmokers | ACC |

**Supplementary Table S1b.** Studies investigating the neuroanatomical (GM) correlates of behavioral manifestations of cognitive impulsivity (risky decision making and impulsive choice tasks)

| **Study** | **Study type** | **Objective** | **ROIs** | **Cognitive impulsivity subdomains** |
| --- | --- | --- | --- | --- |
| Dalley & Robbins (2017) | Literature review of lesion, MRI, and fMRI studies | To investigate the neuroanatimical correlates of cognitive impulsivity | VLPFC, DLPFC, lOFC,  Caudate, Putamen, Ventral striatum | Impulsive choice |
|  |  |  | ACC, VMPFC,mOFC,  Caudate, Putamen, Ventral striatum | Risky decision making |
| Tolomeo et al (2016) | Cross-sectional VBM study | To assess the correlation between GM reductions in opioid dependent individuals* and behavioral manifestations  of cognitive impulsivity as assessed by the CGT | mOFC, Globus pallidus | Risky decision making |
| Durazzo, Meyeroff, and Yoder (2018) | Cross-sectional VBM study | To assess the correlation between GM reductions in chronic smokers and  behavioral manifestations of | ACC, VMPFC | Risky decision making |

|  |  | cognitive impulsivity as assessed by the IGT | 32 |  |
| --- | --- | --- | --- | --- |
| Meade et al (2020) | Cross-sectional VBM study | To assess the correlation between GM reductions in cocaine dependent individuals***** and behavioral manifestations of cognitive impulsivity as assessed by the  Monetary Choice Questionnaire (MCQ) | mOFC, posterior parietal cortex | Impulsive choice |
| Wang et al (2016) | Cross-sectional VBM study | To assess the correlation between GM reductions in individuals affected by alcohol use disorder***** and behavioral manifestations of cognitive impulsivity as assessed by a  Delay Discounting Task (DDT) | VMPFC, ACC, Putamen | Impulsive choice |
| **Note*.** Substance dependent individuals (cocaine, alcohol, opioids) reported a chronic use of tobacco; ROIs= Regions of Interest | | | | |

**Bibliography**

Yang Z, Zhang Y, Cheng J, Zheng RJEJoR. Meta-analysis of brain gray matter changes in chronic smokers. Eur J Radiol 2020;132:109300. <https://doi.org/10.1016/j.ejrad.2020.109300>

Sutherland MT, Riedel MC, Flannery JS, et al. Chronic cigarette smoking is linked with structural alterations in brain regions showing acute nicotinic drug-induced functional modulations. Behav Brain Funct. 2016;12(1):16. Published 2016 Jun 2. doi:10.1186/s12993-016-0100-5

Zhong J, Shi H, Shen Y, et al. Voxelwise meta-analysis of gray matter anomalies in chronic cigarette smokers. Behav Brain Res. 2016;311:39-45. doi:10.1016/j.bbr.2016.05.016

Pan P, Shi H, Zhong J, et al. Chronic smoking and brain gray matter changes: evidence from meta-analysis of voxel-based morphometry studies. Neurol Sci. 2013;34(6):813-817. doi:10.1007/s10072-012-1256-x

Dalley JW, Robbins TWJNRN. Fractionating impulsivity: neuropsychiatric implications. Nat Neurosci 2017;18(3):158. https://doi.org/10.1038/nrn.2017.8

Tolomeo S, Gray S, Matthews K, Steele J, Baldacchino AJPm. Multifaceted impairments in impulsivity and brain structural abnormalities in opioid dependence and abstinence. 2016. Psychol Med https://doi.org/10.1017/S0033291716001513

Durazzo TC, Meyerhoff DJ, Yoder KKJD, dependence a. Cigarette smoking is associated with cortical thinning in anterior frontal regions, insula and regions showing atrophy in early Alzheimer’sDisease.DrugAlcoholDepend2018;192:277-84. https://doi.org/10.1016/j.drugalcdep.2018.08.009

Meade CS, Bell RP, Towe SL, Hall SAJD, Dependence A. Cocaine-related alterations in fronto-parietal gray matter volume correlate with trait and behavioral impulsivity. Drug Alcohol Depend 2020;206:107757. https://doi.org/10.1016/j.drugalcdep.2019.107757

Wang J, Fan Y, Dong Y, Ma M, Ma Y, Dong Y, et al. Alterations in brain structure and functional connectivity in alcohol dependent patients and possible association with impulsivity. PLoS One 2016;11(8):e0161956. https://doi.org/10.1371/journal.pone.0161956
