# Supplementary material for: Neuroanatomical Correlates of Impulsive Choices and Risky Decision Making in Young Chronic Tobacco Smokers: A Voxel-Based Morphometry Study
Source: Front Psychiatry. 2021 Aug 30;12:708925. doi: 10.3389/fpsyt.2021.708925 (PMC8435625; doi:10.3389/fpsyt.2021.708925)
Supplement: Supplementary file 2 [file Table_2.docx]

**Supplementary Table S2.** Inclusion and exclusion criteria

| **Chronic tobacco smokers** | **Non-smokers** |
| --- | --- |
| ***Inclusion criteria*** | ***Inclusion criteria*** |
| Individuals smoking 10 or more cigarettes per day since two or more years | Individuals who never smoked/used tobacco and/or nicotine products |
| Age range 18-50 years old | Age range 18-50 years old |
| Ability to understand English and have the capacity to provide informed consent | Ability to understand English and have the capacity to provide informed consent |
| Currently not enrolled in any smoking cessation program and not taking any pharmacotherapy to aid smoking cessation | N/A |
| CO ≥ 10 ppm | CO ≤ 4ppm |
| Positive to salivary Cotinine (≥20ng/ml) | Negative to salivary Cotinine (<20ng/ml) |
| ***Exclusion criteria*** | ***Exclusion criteria*** |
| Pregnancy | Pregnancy |
| A score < 3 on the FTND  Individuals consuming nicotine through alternative products of nicotine administration (e.g. vaping), and/or smokeless tobacco (e.g. snuff) | N/A  N/A |
| N/A | Ex-smokers |
| individuals with current and/or past licit and/or illicit polysubstance use and dependence* | Individuals with current or past licit and/or illicit polysubstance use and dependence |
| Individuals consuming more than 14 units of alcohol per week | Individuals consuming more than 14 units of alcohol per week |
| Individuals diagnosed with AXIS 1 psychiatric disorder as defined in DSM-V (except Tobacco Use Disorder) | Individuals diagnosed with AXIS 1 psychiatric disorder as defined in DSM-V (except Tobacco Use Disorder) |
| individuals with a history of serious head injury | Individuals with a history of serious head injury |
| Individuals affected by chronic communicable and non-communicable conditions (HIV, Diabetes) | Individuals affected by chronic communicable and non-communicable conditions (HIV, Diabetes) |
| Individuals with metal implants (for MRI purposes) | Individuals with metal implants (for MRI purposes) |
| Individuals with a neurological disorder (including Dementia) | Individuals with neurological disorder including Dementia |
| Individuals presenting with DSM-V acute confusional state | Individuals presenting with DSM-V acute confusional state |
|  |  |
| **Note**. * excluding individuals smoking cannabis recreationally mixed with tobacco; CO=Carbon Monoxide; MRI=Magnetic Resonance Imaging, FTND= Fagerström Test for Nicotine Dependence. ppm= parts per million; ng/ml= nanograms per milliliter;DSM-V= Diagnostic statistical manual of mental disorders version 5. | |
