# Supplementary material for: Neuroanatomical Correlates of Impulsive Choices and Risky Decision Making in Young Chronic Tobacco Smokers: A Voxel-Based Morphometry Study
Source: Front Psychiatry. 2021 Aug 30;12:708925. doi: 10.3389/fpsyt.2021.708925 (PMC8435625; doi:10.3389/fpsyt.2021.708925)
Supplement: Supplementary file 3 [file Table_3.docx]

| **Supplementary Table S3.** Brain structures presenting GM volume reductions in chronic tobacco smokers compared to nonsmokers while controlling for TIV, age, and biological sex. | | | | | | |
| --- | --- | --- | --- | --- | --- | --- |
| **Cluster Index** | **Cluster size (voxels)** | **Region** | **Hemisphere** | **MNI coordinates (x,y,z)** | **BA** | **Peak T value** |
| 1 | 3361 | Cerebellum | L | -30, -75, -30 | N/A | 5.04*** |
|  |  | Cerebellum | L | -29, -66, - 20 | N/A | 3.40** |
|  |  | Cerebellum | L | -23, -51, -14 | N/A | 2.33* |
| 2 | 1938 | Temporal gyrus | R | 44, 15, -27 | 38 | 4.19*** |
|  |  | Temporal gyrus | R | 30, 17, -36 | 38 | 3.31** |
| 3 | 1849 | Cingulate gyrus | R | 12, 6, 38 | 24 | 4.13*** |
|  |  | Cingulate gyrus | R | 9, -11, 30 | 23 | 3.47** |
|  |  | Cingulate gyrus | R | 14, 21, 29 | 32 | 3.28** |
| 4 | 3099 | Middle temporal gyrus | L | -51, -69, 6 | 39 | 3.72*** |
|  |  | Inferior temporal gyrus | L | -48, -71, 6 | 37 | 3.14** |
|  |  | Superior occipital gyrus | L | -38, -78, 24 | 19 | 2.91** |
| 5 | 284 | Middle frontal gyrus | R | 42, 0, 39 | 6 | 3.69*** |
|  |  | Middle frontal gyrus | R | 35, -8, 42 | 6 | 2.84** |
| 6 | 743 | Medial frontal gyrus | L | -21, 41, 23 | 9 | 3.60*** |
|  |  | Inferior frontal gyrus | L | -39, 41, 3 | 46 | 2.43* |
|  |  | Inferior frontal gyrus | L | -27, 38, 11 | 46 | 2.12* |
| 7 | 281 | Postcentral gyrus | R | 60, -5, 12 | 43 | 3.42** |
|  |  | Precentral gyrus | R | 57, 6, 8 | 6 | 2.69** |
| 8 | 462 | Posterior cingulate | L | -23, -62, 12 | 31 | 3.31** |
| 9 | 151 | Middle frontal gyrus | L | -23, 6, 42 | 6 | 3.14** |
| 10 | 265 | Middle frontal gyrus | L | -35, 47, -8 | 11 | 2.82** |
|  |  | Inferior frontal gyrus | L | -26, 35, -12 | 47 | 2.70** |
| 11 |  | Cingulate gyrus | L | -11, 26, 18 | 32 | 2.07** |
| 12 | 736 | Superior temporal gyrus | L | -45, 11, -26 | 38 | 2.56** |
|  |  | Uncus | L | -24,17, -36 | 38 | 2.33* |
| 13 |  | Superior temporal gyrus | L | -33, 21, -29 | 38 | 2.00* |
| 14 | 223 | Caudate | L | -11, 2, 12 | N/A | 2.43* |
|  |  | Putamen | L | -21, -9, 6 | N/A | 1.89* |
| 15 | 143 | Middle frontal gyrus | L | -26, -11, 35 | 8 | 2.22* |
| 16 | 256 | Orbital gyrus | L | -5, 44, -27 | 11 | 1.91* |
| **Note.** *** *p*<0.0001,** *p*<0.01, **p*<0.05. The cluster-forming threshold consisted in *p*<0.05 with a minimum of 100 contiguous voxels per cluster. R= Right; L=Left; MNI= Montreal Neurological Institute; T=t-statistic; BA= Brodmann’s area. | | | | | | |
