# Supplementary material for: Neuroanatomical Correlates of Impulsive Choices and Risky Decision Making in Young Chronic Tobacco Smokers: A Voxel-Based Morphometry Study
Source: Front Psychiatry. 2021 Aug 30;12:708925. doi: 10.3389/fpsyt.2021.708925 (PMC8435625; doi:10.3389/fpsyt.2021.708925)
Supplement: Supplementary file 4 [file Table_4.docx]

| **Supplementary Table S4.** Voxel-wise regression results depicting significant positive associations between GM volume in brain regions of no interest and impulsive choices while controlling for TIV, age, and biological sex. | | | | | | | | |
| --- | --- | --- | --- | --- | --- | --- | --- | --- |
|  | **Brain region** | **Hemisphere** | **BA** | **MNI**  **coordinates (x,y,z)** | **Peak T**  **values** | **P**  **values** | **Cluster size (k)** | **R^2^** |
| Impulsive choice (ED50 values) | Supplementary motor area | L | 6 | -6, -12 ,72 | 5.01 | *p*<0.0001 | 4744 | 0.404 |
|  |  | R | 6 | 9, -5, 72 | 3.70 | *p*<0.005 |  | 0.309 |
|  | Precuneus | L | 7 | -24, -63, 26 | 3.86 | *P*<0.005 | 280 | 0.306 |
|  | Inferior occipital gyrus | L |  | -33, -98, -17 | 3.48 | *p*<0.005 | 1176 | 0.289 |
|  | Lingual gyrus | L | 18 | -17,-75,5 | 3.37 | *P*<0.005 | 267 | 0.263 |
|  | Para-hippocampal gyrus | R | 36 | 44, -30, -18 | 2.63 | *p*<0.01 | 132 | 0.188 |
|  | Cerebellum | L | N/A | -5,-45, -11 | 2.50 | *P*<0.05 | 252 | 0.175 |
|  | Post-central gyrus | L | 3 | -38,-30, 59 | 2.35 | *P*<0.05 | 169 | 0.159 |
| **Note.** BA= Brodmann Area; MNI= Montreal Neurological Institute; ED50= effective delay 50 ; R2=coefficient of determination. The cluster forming threshold consisted in p<0.05 with a minimum of 100 contiguous voxels per cluster. | | | | | | | | |
