# Supplementary material for: Neuroanatomical Correlates of Impulsive Choices and Risky Decision Making in Young Chronic Tobacco Smokers: A Voxel-Based Morphometry Study
Source: Front Psychiatry. 2021 Aug 30;12:708925. doi: 10.3389/fpsyt.2021.708925 (PMC8435625; doi:10.3389/fpsyt.2021.708925)
Supplement: Supplementary file 5 [file Table_5.docx]

| **Supplementary Table S5.** Voxel-wise regression results depicting significant negative associations between GM volume in brain regions of no interest, risk taking, and overall proportion bet scores while controlling for TIV, age, and biological sex. | | | | | | | | |
| --- | --- | --- | --- | --- | --- | --- | --- | --- |
|  | **Brain region** | **Hemisphere** | **BA** | **MNI**  **coordinates (x,y,z)** | **Peak T**  **values** | **P**  **values** | **Cluster size (k)** | **R^2^** |
|  | Middle frontal gyrus | R | 10 | 33,47,9 | 4.84 | P<0.0001 | 1065 | 0.546 |
| Risk taking | Para-hippocampal gyrus (uncus) | L | 36 | -24, 3, -30 | 4.59 | *P*<0.0001 | 335 | 0.525 |
|  | Para-hippocampal gyrus | R | 36 | 39, -27, -21 | 3.91 | *P*<0.005 | 659 | 0.446 |
|  | Cerebellum | L | N/A | -18, -75, -20 | 4.46 | *P*<0.0001 | 1402 | 0.563 |
|  | Fusiform gyrus | L | 19 | -44, -68, 14 | 3.73 | *P*<0.005 | 461 | 0.426 |
|  | Superior temporal gyrus | L | 38 | -51, 0, -18 | 3.53 | *P*<0.005 | 190 | 0.406 |
|  | Middle frontal gyrus | L | 6 | -21, -21, 62 | 3.50 | *P*<0.005 | 748 | 0.392 |
|  | Precentral gyrus | R | 4 | 30, -32, 65 | 3.36 | *P*<0.005 | 2140 | 0.373 |
|  |  |  |  |  |  |  |  |  |
| Overall proportion bet | Cerebellum | L | N/A | -18, -75, -20 | 4.63 | *P*<0.0001 | 1136 | 0.583 |
|  | Middle frontal gyrus | R | 10 | 33,47, 9 | 4.38 | *P<*0.0001 | 827 | 0.268 |
|  | Para-hippocampal gyrus | L | 36 | -24, 3, -30 | 3.99 | *P*<0.0001 | 259 | 0.457 |
|  | Para-hippocampal gyrus | R | 36 | 39, -27, -21 | 3.70 | *P*<0.005 | 210 | 0.419 |
|  | Middle temporal gyrus | R | 39 | 44, -68, 9 | 3.50 | *P*<0.005 | 117 | 0.391 |
|  | Middle temporal gyrus | L | 37 | -53, -53, -3 | 3.32 | *P*<0.005 | 124 | 0.367 |
|  | Superior frontal gyrus | L | 6 | -15, 11, 57 | 3.34 | *P*<0.005 | 421 | 0.370 |
|  | Occipital gyrus | L | 19 | -41, -80, 5 | 3.23 | *P*<0.005 | 318 | 0.355 |
|  | Superior temporal gyrus | R | 39 | 42, -53, 23 | 3.18 | *P*<0.005 | 315 | 0.347 |
|  | Superior temporal gyrus | L | 36 | -51, 0, -18 | 3.18 | P<0.005 | 112 | 0.357 |

**Note.** BA= Brodmann Area; MNI=Montreal Neurological Institute; R^2^=Coefficient of determination. The cluster forming threshold consisted in p<0.05 with a minimum of 100 contiguous voxels per cluster.
