# Supplementary material for: Neuroanatomical Correlates of Impulsive Choices and Risky Decision Making in Young Chronic Tobacco Smokers: A Voxel-Based Morphometry Study
Source: Front Psychiatry. 2021 Aug 30;12:708925. doi: 10.3389/fpsyt.2021.708925 (PMC8435625; doi:10.3389/fpsyt.2021.708925)
Supplement: Supplementary file 6 [file Table_6.docx]

| **Supplementary Table S6.** Voxel-wise regression results depicting associations between brain regions of no interest and tobacco smoking variables while controlling for TIV, age, and biological sex | | | | | | | | |
| --- | --- | --- | --- | --- | --- | --- | --- | --- |
| **Pack years** | **Brain region** | **Hemisphere** | **BA** | **MNI**  **coordinates (x,y,z)** | **Peak T**  **values** | **P**  **values** | **Cluster size (k)** | **R^2^** |
|  | Middle frontal gyrus | L | 6 | -14, -3, 56 | 4.57 | *P*<0.0001 | 250 | 0.200 |
|  | Parietal supramarginal gyrus | L | 40 | -33, -50, -32 | 4.16 | *P*<0.0001 | 201 | 0.181 |
|  | Transverse temporal gyrus | L | 41 | -50, -29, 11 | 4.02 | *P*<0.0001 | 107 | 0.149 |
|  | Transverse temporal gyrus | R | 41 | 53, -18, 18 | 4.02 | *P*<0.0001 | 137 | 0.175 |
|  | Paracentral lobule | L | 31 | -6, -24, 51 | 3.42 | *P*<0.005 | 124 | 0.149 |
|  |  |  |  |  |  |  |  |  |
| **Age started smoking** | Paracentral lobule | R | 5 | 14, -35, 57 | 5.73 | *P*<0.0001 | 571 | 0.576 |
|  | Paracentral lobule | L | 6 | -9, -26, 53 | 4.33 | *P*<0.0001 | 175 | 0.453 |
|  | Supramarginal gyrus | R | 40 | 32, -41, 29 | 5.68 | *P*<0.0001 | 800 | 0.437 |
|  | Supramarginal gyrus | L | 40 | -39, -41, 38 | 3.64 | *P*<0.005 | 197 | 0.373 |
|  | Middle occipital gyrus | L | 19 | -29, -87, 21 | 3.50 | *P*<0.005 | 250 | 0.370 |
|  |  |  |  |  |  |  |  |  |
| **Cigarettes smoked x day** | Parietal lobe, Precuneus | L | 31 | -12, -51, 27 | 4.35 | *P*<0.0001 | 342 | 0.336 |
|  |  |  |  |  |  |  |  |  |
| **FTND** | Precuneus | R | 7 | 23, -50, 47 | 4.72 | *P*<0.0001 | 478 | 0.372 |
|  | Precentral gyrus | R | 6 | 41, -6, 38 | 4.56 | *P*<0.0001 | 102 | 0.361 |
|  | Posterior Cingulate Gyrus | R | 31 | 18, -32, 47 | 4.29 | *P*<0.0001 | 107 | 0.347 |
|  | Middle occipital gyrus | R | 19 | 53, -75, -11 | 3.47 | *P*<0.005 | 1092 | 0.270 |
|  |  |  |  |  |  |  |  |  |
| **Note.** The cluster forming threshold consisted in *p*<0.01 (unc.) with a minimum of 100 contiguous voxels per cluster. R^2^= coefficient of determination; FTND= Fagerström Test for Nicotine Dependence | | | | | | | | |
