# Supplementary figures and images for: Neuroanatomical Correlates of Impulsive Choices and Risky Decision Making in Young Chronic Tobacco Smokers: A Voxel-Based Morphometry Study
Source: Front Psychiatry. 2021 Aug 30;12:708925. doi: 10.3389/fpsyt.2021.708925 (PMC8435625; doi:10.3389/fpsyt.2021.708925)

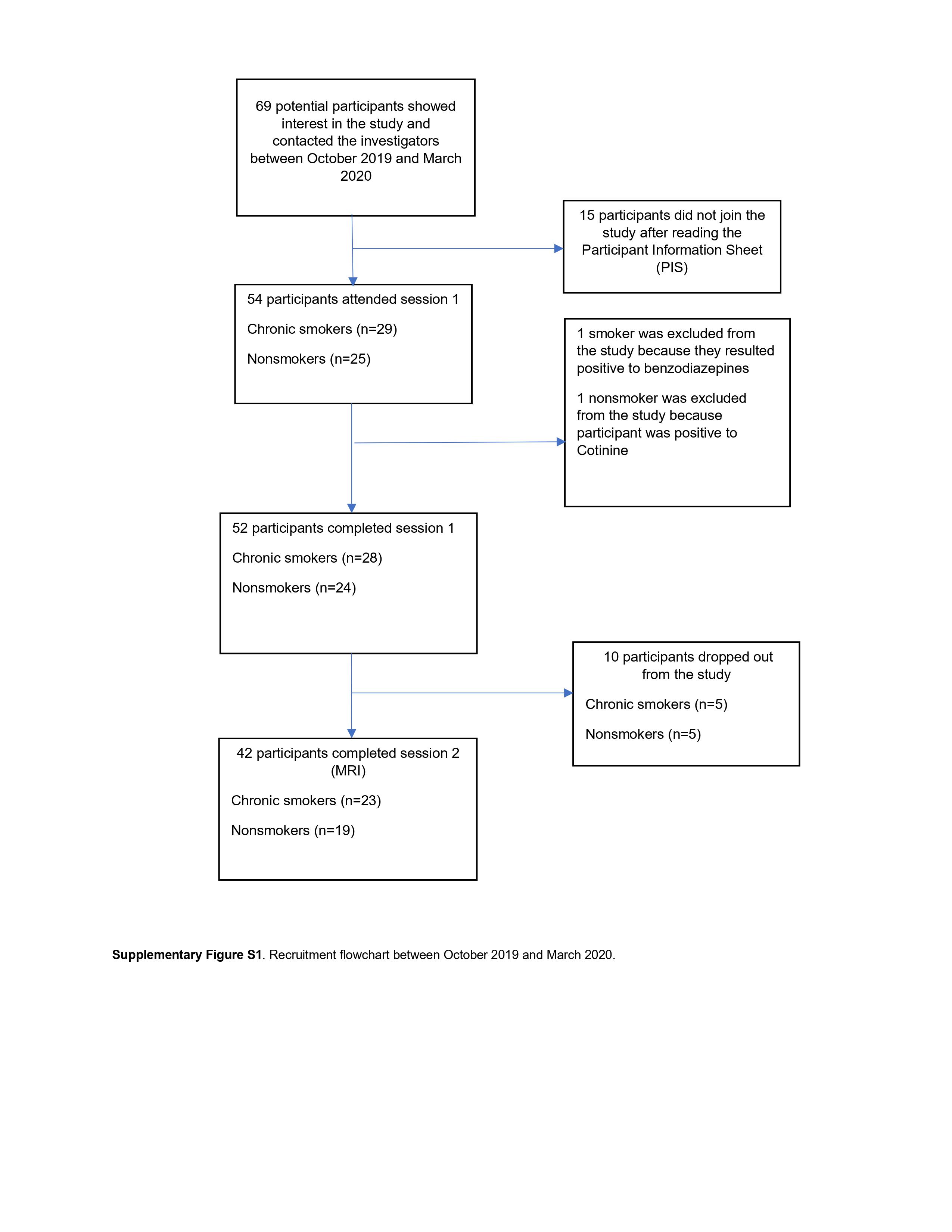

Supplement: Supplementary file 7 [file Image_1.jpg]

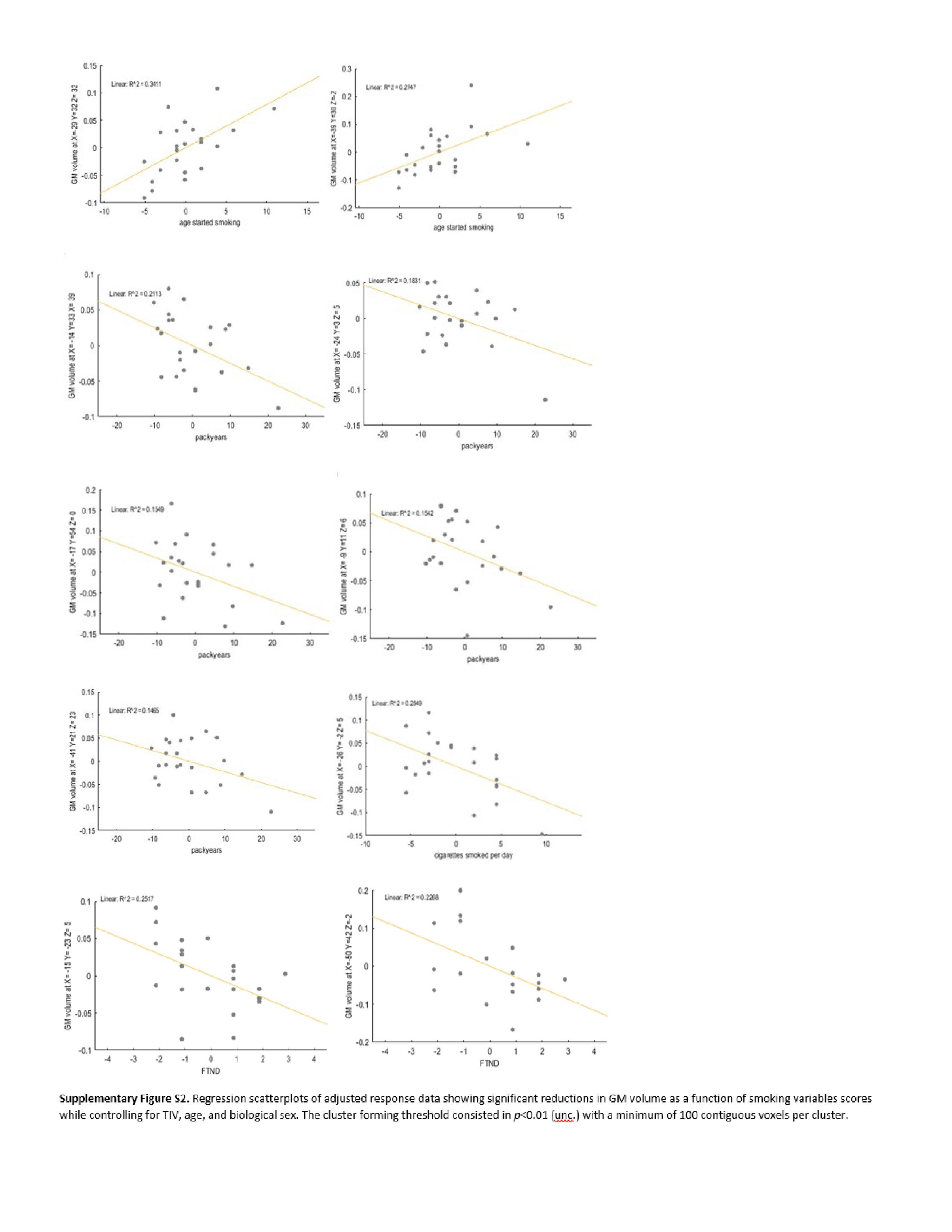

Supplement: Supplementary file 8 [file Image_2.jpg]
